# On the Remote Cause of Epidemic Disease

**Published:** 1842-04

**Authors:** 


### ART. XVI.

*On the Remote Cause of Epidemic Diseases.* By JOHN PARKIN, Graduate of the University of Erlangen, &c. &c.—*London*, 1841. 8vo, pp. 198.

WHEN an author presents to the world views professing to be original, two important questions arise, the one as to their truth, and the other as to their novelty. Dr. Parkin has been at the trouble of writing a book to demonstrate that gases extricated from the bowels of the earth by volcanic action are the remote causes of epidemic diseases. In supposing that there is anything new in this notion, he merely shows that he is little versed in the literature of his subject. Early in the sixteenth century, Horatius Lymbisanus (Orazio Limbisano, M.D.) published a work entitled "De terræ motu prout pestis causa est disputatio," (4to, Neapoli, 1629.) What his particular opinions were we do not know, having never been able to get a sight of the book; nor do we think it needful to hunt through the numerous writers who have alluded to the subject in one way or other. For Dr. Parkin's edification, however, we will quote the following passage from an author who is, or ought to be, very well known—*Sennertus.*

"Verum præter cœlum, aliæ etiam plures causæ dantur, à quibus aëri pestilentia communicatur, atque ipse inde pestilens redditur. Primo scilicet aër venenatus et pestilens redditur, cum venenatæ in infimis terræ cavernis diu contentæ auræ copiosius aëri permiscentur. Halitus illi venenati gignuntur ex aëre diu concluso, et venenatis auris metallicis mixto, stagnantibus aquis alicubi conclusis et putrescentibus. Commoventur autem illi halitus venenati iisque exitus sæpe patefit à terræ motu; extantque passim in historiis exempla terræ motuum quos gravissimæ pestes secutæ sunt." (De Febribus, Lib. iv. c. 2.)

This, it will be observed, is almost to an iota the opinion of Dr. Parkin as expressed in chapter iii. of his work. It is singular, also, that Dr. Parkin should not have thought Noah Webster, an author whom he quotes with reference to other matters, worthy of mention in connexion with the supposed volcanic origin of epidemic diseases, for this writer labours hard to establish a general relation among epidemics, volcanoes, earthquakes, and comets, and attempts in several instances to show the connexion between particular epidemics and particular earthquakes, although he is doubtful whether the intemperaments of nature produce disease directly by their action on the living body, or indirectly by blighting the products of vegetation, and thence deteriorating the chief articles of food.*

* A Brief History of Epidemic and Pestilential Diseases, &c. 2 vols. 8vo.—Hartford, 1799.

So much for the novelty of the volcanic hypothesis: let us now enquire into the validity of the arguments adduced by Dr. Parkin in its support. To entertain this hypothesis, even for a moment, it must of course be admitted that the spread of all epidemics observes laws nearly or entirely similar. Now our author's remarks throughout the volume relate principally to *cholera*, and he seems to take it for granted that this disease will serve as a type of epidemics in general. We allow that there are some points of analogy between the course and mode of extension of cholera and influenza, but with respect to most epidemics we can see no features in common, except their attacking multitudes of people, visiting various regions of the earth, and being frequently preceded or accompanied by unusual conditions of the atmosphere—grounds surely insufficient for referring them to a common law or a similar agency. Granting, however, for the sake of argument, that one epidemic will serve as a type of all the rest, we will proceed to examine the reasoning by which Dr. Parkin endeavours to carry out his analogy between the cause of epidemics and volcanic action.

"If," says he, "we generalize the phenomena attendant on the march of epidemics, we shall find that they are so regular and uniform as to deserve to be set down as the laws of the disease. More than this, if we compare these laws with those attendant on volcanic action, we shall find that they are the same or similar, as will be apparent by the recital of a few of the principal phenomena observed during the operation of this process on the crust of the globe." (p. 39.)

1. The first coincidence stated by Dr. Parkin is, that the effects of volcanic action are observed along particular lines of the earth's surface, and that the same is the case with respect to epidemics, whence a striking analogy between the two.

Now, it is evident that the mere fact of epidemics following particular lines on the earth's surface, would no more establish their connexion with volcanic action than with the Great Western Railway, or anything else that observes a particular line on the earth's surface, unless it could be shown that volcanic actions and epidemic diseases observed the *same*, or *corresponding* lines. This is not asserted by Dr. Parkin in his illustration of the law just announced, but further on he does actually venture on such a statement with reference to the *black death* and the *cholera*.

"Simultaneously with the commencement of these diseases, there were experienced severe and remarkable concussions of the earth, as well as other signs of the existence of volcanic action; and which gradually extended over Asia and Europe, in the same direction and along the same lines as those taken by the diseases themselves." (p. 48.)

That volcanic phenomena, and natural convulsions of all kinds, were extraordinarily frequent during the prevalence of the black death is very well known; since the cholera made its appearance in 1817, there has been an average of volcanic action throughout the world not greater than that observed during the same number of years at most other periods —indeed we rather think less, but will not insist on the point, as we have not leisure to investigate it properly: that either the black death, or the cholera, observed the same lines as the volcanic actions contemporaneous with them, is one of the most bare-faced assertions we have met with for some time.

With respect to the black death, our author adduces sufficient evidence of the prevalence of the volcanic phenomena over the world in general, simultaneously with that of the pestilence; but there is not a syllable to identify the lines in which they moved. With respect to the cholera, he shows that a short time after its great epidemic eruption in India, several shocks of earthquakes were experienced in different parts of Bengal; but we are not informed that the course of the disease bore any particular geographical relation to the course of the subterraneous agency. In the year 1819, a more violent earthquake than had before been known in India, shook the Peninsula, but we still hear nothing of the *lines,* and between the years 1817 and 1819, the cholera seems to have been moving in various directions, and doing its work with uncommon fury, without any appreciable volcanic assistance. In 1821 the epidemic marched into Persia, but no earthquake is to be met with till 1824, and even here there is no coincidence of places. Once for all we ask *where are the lines?* It would evidently be useless to follow Dr. Parkin and the cholera into Europe, where all we meet with about earthquakes is that they "were felt after the appearance of the disease in Odessa, in Northern Europe, and along the Rhine, while even in England, where such phenomena are almost unknown, slight shocks have been felt occasionally from that time to the present." (p. 140.) For the rest our author is obliged to have recourse to meteorological phenomena, and atmospheric vicissitudes, which he *presumes* to be connected with volcanic action.

2. Another law, which, according to our author, is characteristic alike of volcanic action and epidemic influence, is the regularity of their progress, both chronologically and geographically. In his remarks on this law Dr. Parkin treats us to a *lucus à non lucendo;* for he admits that "this rule is not so evident with regard to the formation of volcanic vents on account of the want of historical data in such instances, their production being frequently the work of ages, it is yet sufficiently clear to cause it to be set down as one of the laws of volcanic action; for the vents along a particular line are not formed at once, but in succession;" in the same strain he adds that the minor effects of the same cause, earthquakes to wit, afford better illustration of the progressiveness of volcanic action; and the upshot of the whole appears to be that since volcanic actions have been observed at successive periods, and in different places, their occurrence is therefore *chronologically and geographically regular,* and that a similar regularity in the occurrence of epidemic diseases binds them in the most intimate relations with volcanic actions. (pp. 47-8.)

It is quite clear that all sublunary events must occur either simultaneously, or in succession, and in the same place or in different places; and Dr. Parkin's reasoning, therefore, applies no more to the subject in hand than to any other in which succession and progression are concerned.

3. According to Dr. Parkin, a third law of subterranean action is, that its effects are less on secondary than on tertiary strata, while they are seldom witnessed on primary formations; and the same law applies, with equal force, to the march of epidemic diseases, which is most frequent over tracts of tertiary formation, comparatively rare on secondary strata, and almost unknown in districts resting on primary formations.

Our author, on the whole, is correct in this statement, with respect to volcanic action as now exhibited on the earth's surface; but in extending it to epidemic diseases he is merely indulging in one of those hasty generalizations in which he seems so greatly to delight. As usual, he appeals to *cholera*, but in the present state of geological knowledge we are in no condition to speculate on the relations which this or any other extensive epidemic may bear to the geological characters of the countries over which it prevails. Thus Asia has been the cradle of various pestilences, which have swept for thousands of miles over its surface before reaching the other quarters of the globe; but the geology of Asia is yet very imperfectly known, and we are convinced that if we were to point out the course of an epidemic over Asia to Buckland or Conybeare, and ask what were the geological characters of all the districts through which it passed, they would at once declare that they did not know. Of a great part of the interior of Africa neither the geology nor the diseases are known; and if Dr. Parkin is acquainted with the geology of every part of America, we suspect he knows considerably more of the matter than anybody else. In favour of the connexion between epidemics and recent geological formations, Dr. Parkin adduces the well-known fact that *cholera* is observed to be frequent along sea shores and the banks of rivers ; but we are not aware that this obtains generally with respect to other epidemics, and even with respect to cholera it argues quite as much on the side of low and moist localities as of tertiary strata. The best evidence in favour of his geological law adduced by Dr. Parkin is the course taken by the cholera on its first entrance into Europe.

> "It will be seen," he says, "that the tract of country traversed by the epidemic with such rapidity forms one single and immense tertiary deposit; being bounded to the north by a chain of primary mountains through a great part of its extent. This barrier here, as elsewhere, was sufficient to prevent the spread of the disease in that direction; for, although extending along the whole of this plain from east to west, the disease was not seen on the southern side of this range of mountains—as Switzerland, which is situated in the midst of the chain, not only escaped then, but has, I believe, continued free from any visitation of the epidemic up to the present time," &c. (p. 55.)

But these observations, though specious, have no real weight. Doubtless they illustrate the influence of *locality*, but they leave the geological question untouched, as will be evident when we consider, on the one hand, that extensive levels are never to be found on primary formations, and, on the other, that mountain ranges of any elevation consist, for the most part, of primary or transition rocks. All, therefore, that Dr. Parkin's observations go to prove is, that cholera traverses plains easily, but does not readily ascend mountains ; which is just what might be expected, and what has been repeatedly observed with regard to epidemics in general.

4. The fourth law stated by Dr. Parkin is, that the effects of volcanic action are much greater and more perceptible near the sea and other large collections of water, as lakes, rivers, &c. and that the same obtains with respect to epidemic diseases. We believe this law, as a general one, may be admitted with reference to volcanic action. In applying it to disease, our author betakes himself once more to his favorite *cholera.* No doubt this disease, for some reason which we attempt not to explain, has a disposition to follow water-courses, but it has also been frequent and severe where there was little water. With regard to other epidemics, as typhus, influenza, &c., we are quite unaware of any particular relation to water, except in as far as low and damp situations are unhealthy, and may therefore be favorable to the production of many diseases.

Dr. Parkin errs greatly if he supposes that any point in science can be established by such vague generalities as these, which are in effect not worth arguing upon. In all such cases the *onus probandi* rests with the assertor, and we shall think it time enough to consider the nature of the relation borne by epidemics in general to the sea, lakes, and rivers, when some evidence of the existence of such relation beyond the mere assertion is brought forward.

5. The last law common to volcanic actions and epidemic causes on which we have to comment is "their limited duration, their periodical return, and their total cessation in that particular locality after certain definite periods." (p. 60.) Dr. Parkin's illustrations of this law are exceedingly meager, and for a very plain reason, namely, the extreme dearth of materials. With regard to volcanic actions in particular localities having a limited duration and an end, this simply places them in the same category with everything else which is neither eternal nor immortal. With regard to their *periodicity,* all the evidence offered by our author is contained in the following passage.

"Volcanoes only throw out lava for a short period, as a few hours, or a few days, although the minor products of the same process, or aqueous vapour and gaseous substances, continue to be evolved for a much longer time, as several months. When, however, a vent has been once formed in any locality, eruptions are sure to be experienced from time to time, in the same spot, although the period of their return varies much in different situations. The same circumstance is observed with regard to earthquakes, except that they return more frequently and at shorter intervals than the eruption of the volcano; for although the duration of a single shock is seldom more than a few minutes, still a succession of shocks is sometimes felt in the same spot for many weeks, or even months. They then subside entirely for a period which varies under different circumstances, when they again return and again subside, to reappear after another interval; for the same continuous tracts, as Lyell justly observes, are agitated again and again. In some situations they are found to return at regular and fixed periods or months in the year, more particularly the summer season." (pp. 60-1.)

Dr. Parkin's remarks on the corresponding periodicity of epidemics are of the same feeble and unsatisfactory character. With respect to the final cessation of epidemics in the places where they originally appeared, we cannot conceive what has led him to an assertion so obviously in the face of all fact. The plague still exists in the places where it was first known; the cholera still exists in India; we know not in what part of the east smallpox and measles originated, but they exist all over the world: in short, it is needless to increase the catalogue of epidemics which have continued from time to time to infest the country of their birth; for all the more important epidemics have done so, except the sweating sickness and the black death, the latter of which can hardly be considered as a distinct disease, having evidently been nothing more than an intense form of plague, complicated with typhoid pneumonia. The black death, however, is the only instance adduced by our author in support of his sweeping assertion. He is of opinion that this disease, or one similar to it, exists in the present day. Without stopping to enquire into the truth of this assertion, we think this the right time for asking a question which suggested itself the moment we took up Dr. Parkin's book, but which we delayed proposing in order that we might examine all his laws and give his arguments fair play. It is this: If volcanic action be the cause of epidemic disease, how is it that nothing is laid to the charge of Hecla, one of the most indefatigable of volcanoes? Hecla is many hundred miles nearer to Scotland and Norway than Ætna or Vesuvius to Egypt and Syria; why, therefore, are the two former countries not perpetually ravaged by the plague or some other pestilence? We might also ask, why should Aleppo or Cairo, situated more than a thousand miles from Ætna or Vesuvius, suffer so much more from their pestilential influence than Naples or Catania, situated at their very base? We fear that these and a few similar questions, rigorously pushed home, would reduce the volcanic hypothesis to a very helpless condition. But it is time to bring our strictures to a close. Before doing so, however, we should add, that our author regards endemic diseases as originating from the same volcanic source as epidemic diseases. This he argues from two alleged circumstances: First, that all endemic maladies have at some former period been epidemic; and secondly, that they prevail in their greatest extent and intensity in those regions where volcanic action is most manifest. As on former occasions, one or two feeble instances serve to establish these positions satisfactorily to our author's mind; that they are, however, altogether erroneous will be very easily perceived. When has cretinism or Barbadoes leg been epidemic? And what have the pestiferous vapours breathing from the swamps of the African rivers to do with volcanic action?

Dr. Parkin comments at some length on the natural history of volcanic operations; and on the whole he seems to make a better figure as a geologist than as an etiologist of epidemic and endemic diseases: this part of the subject, however, is not within our province. Although the foregoing hypothesis has received very poor support from the arguments of Dr. Parkin, we would not be supposed to reject it as altogether unworthy of consideration. With respect to one epidemic, cholera, the fact of its advancing rapidly in opposition to the strongest winds is much in favour of the belief that it is propagated by some cause operating beneath the earth's surface—whether by the extrication of miasmata, or by some change in the electricity or other properties of the surface; nor is it impossible that such cause may participate in the nature of volcanic action. The question is evidently one to be kept in view, but certainly not one to be solved in the present state of science.

